# Does Students’ Level of Intelligence Moderate the Relationship Between Socio-Economic Status and Academic Achievement?

**DOI:** 10.3390/jintelligence12120123

**Published:** 2024-12-01

**Authors:** Ricarda Steinmayr, Ursula Kessels

**Affiliations:** 1 Institute of Psychology, Technische Universität Dortmund, 44227 Dortmund, Germany; 2 Department of Education and Psychology, Freie Universität Berlin, 14195 Berlin, Germany; ursula.kessels@fu-berlin.de

**Keywords:** intelligence, socio-economic status, grades, math competencies, reading competencies

## Abstract

The association between school students’ social background and school achievement is well documented. Recent studies demonstrated that this association might be moderated by the level of cognitive potential. Based on these results, we recruited an elementary school sample (*N* = 837) and an adolescent sample at the end of their compulsory school time (*N* = 2100) to investigate whether the associations between students’ social background and their academic achievement in math and language arts were moderated by the level of their general cognitive competencies, i.e., intelligence. To this end, we assessed intelligence, math and reading competencies, teacher-reported grades, and students’ socio-economic background (number of books at home). In both samples, the association between students’ socio-economic background and language arts grades was moderated by their intelligence level but not the ones with math grades, reading, and math competencies. The association between socio-economic background and language arts grades was strongest in the average intelligence sample and smaller in the above-average intelligence sample. Results are discussed with regard to their implications for the discussion of social injustice in schools.

## 1. Introduction

Academic achievement plays an extremely important role in students’ lives, as grades and performance in academic achievement tests predict future success in educational and occupational contexts (Borghans et al. 2016; Byrnes and Miller 2007; Mattern et al. 2013) and are frequently used for selection and admission purposes throughout their academic career (Burton and Ramist 2001; Mattern et al. 2013). It is a societal problem that both indicators are highly associated with students’ social background (grades: e.g., Johnson et al. 2007; Steinmayr et al. 2012; academic achievement tests: e.g., OECD 2016), as it prevents socially disadvantaged students from having equal academic chances. This problem is even more severe than it would seem on the surface, as even students with lower social background displaying equal levels of objectively measured competencies and other important predictors of academic achievement such as intelligence receive worse grades and, in the German tracked school system, lower transition recommendations in their final year of elementary school by their teachers (e.g., Lüdemann and Schwerdt 2013; Steinmayr et al. 2010, 2012). Thus, students with lower social background have worse educational chances independent of their competencies and cognitive potential. As scholastic competencies and cognitive potential are a prerequisite for creative achievements and future educational success (Jauk et al. 2014; Ritchie and Bates 2013), more cognitively competent individuals have better mental prerequisites for inventing and discovering new things, which should ultimately benefit an entire economy. Thus, if schools do not compensate for the worse educational chances of lower SES students, this results in a societally threatening waste of cognitive potential.

However, in these studies, intelligence and competencies were considered as mediators or covariates giving information on social gaps in grades or transition decisions when students objectively performed equally. Thus, they do not give information on whether the association between social background and academic achievement is the same across all intelligence and competency levels. Some intelligence researchers claim that the association between socioeconomic status (SES) and academic achievement can be fully attributed to students’ intelligence (Colom and Flores-Mendoza 2007), which implies that SES and academic achievement would not be related anymore at different levels of intelligence. Other researchers argue that the association between SES and academic achievement is particularly strong at the lower levels of intelligence, as intelligence is an important prerequisite for academic achievement, and high-SES parents have more opportunities to compensate the lack of school-relevant abilities (e.g., Esping-Andersen and Cimentada 2018). Given the linear relationship between intelligence and academic achievement (e.g., Roth et al. 2015), it is also plausible to assume the opposite and expect a weaker association between SES and academic achievement at the lower and higher ends of the intelligence distribution. Among all student characteristics, intelligence is the most important single predictor of academic achievement (Hattie 2008). Thus, at the lower end of the intelligence distribution, a lower level of intelligence should be more difficult to compensate than in the medium-intelligence range. At the higher end of the intelligence distribution, no compensation is necessary.

In the present study, we investigated whether intelligence level moderates the association between social background and two different measures of academic achievement in an elementary school sample before transition and a secondary school sample before transition. We focus on intelligence, as it is known to be the best predictor of several indicators of future success, among them academic achievement (Kuncel et al. 2004). We used objective reading and math competency tests as well as grades as non-objective indicators of academic achievement, as grades precede transition decisions and are the only salient and utilized indicator of academic achievement in some countries (Klieme et al. 2010).

## 2. Theoretical Framework

### 2.1. Socioeconomic Status and Scholastic Achievement

Students’ academic achievement is related to their families’ socioeconomic status. Meta-analyses on the association between SES and academic achievement found an average effect size on the student level of *r* = 0.25 (White 1982) in earlier decades, and more recent studies (1990–2000) found an average effect size of *r* = 0.27 (CI-95%: 0.23–30) (Sirin 2005). In the meta-analyses by White (1982), verbal achievement was more strongly associated with SES than mathematical achievement, while the opposite was found in an analysis by Sirin (2005). As the association between a predictor and academic achievement often relies on the achievement indicator used in the analysis (Lauermann et al. 2020), which was not considered in both meta-analyses, we differentiate between standardized achievement tests and grades in the present study.

At elementary school level, the association between SES and standardized competencies is higher for reading competencies (Hußmann et al. 2017) than for math competencies (Stubbe et al. 2016), whereas at secondary-school level, the association between SES and test performance does not differ between mathematics, reading, and science and is about *r* = 0.35 across all OECD countries (OECD 2016). However, the association between SES and competency tests seems to be stronger in secondary school than in elementary school (cf. OECD 2016 vs. PIRLS: Hußmann et al. 2017; TIMSS: Stubbe et al. 2016; von Stumm and Plomin 2015). At least in Germany, this phenomenon might be partly explained by educational stratification at secondary level. After the fourth grade, students are sorted into different tracks, and tracking seems to reinforce the link between SES and academic achievement assessed via performance tests (Maaz et al. 2008).

As in international meta-analyses (White 1982), the majority of studies from Germany also found SES and grades to be associated. In elementary school, substantial correlations of up to *r* = 0.40 were found between the highest occupational status (HISEI) and grades (Gröhlich and Guill 2009), with slightly higher associations in native language arts grades than in mathematics. At secondary school, the association between SES and grades is smaller, *r* < 0.20, for different subject grades (Steinmayr et al. 2010, 2012). The diminished association between SES and grades in secondary school might also be partly explained by the tracked school system. Students are tracked by both achievement and SES (Maaz et al. 2008), leading to a diminished variance in both variables at one school track. However, as many teachers apply grading on a curve (Calsamiglia and Loviglio 2019), grades, in contrast to achievement tests, only slightly differ between school tracks (see Lauermann et al. 2020).

While the link between family SES and students’ school performance is empirically well established, the reasons for this are less clear. Following Boudon (1974), the overall effect of SES on educational attainment can be separated into primary and secondary effects. Secondary effects comprise the phenomenon that even when controlling for performance, the impact of SES on transition decisions differ by social class, which is explained by social differences in values and expectations (e.g., Erikson and Jonsson 1996; Forster 2021). Primary effects embrace social differences in actual educational performance, e.g., in academic competencies or grades (Boudon 1974), which are partly explained by a wide range of better learning opportunities in higher-SES families. For instance, parental involvement regarding school is higher in high-SES families (Benner et al. 2016; Roksa and Potter 2011), and parental involvement affects their children’s academic achievement more strongly during elementary school than in later years (Jeynes 2005, 2007; Patall et al. 2008). A different explanation focuses intelligence as mediating the link between SES and academic achievement because intelligence is related to both (Lauermann et al. 2020; Roth et al. 2015; Strenze 2007). Studies from Ireland and Brazil found that SES explained almost no variance in academic competencies when intelligence was controlled for (Colom and Flores-Mendoza 2007; O’Connell 2018). However, Steinmayr et al. (2010, 2012) still found an association between grades and SES after controlling for intelligence in German samples (see also Lauermann et al. 2020).

In summary, SES and competency test scores are more strongly correlated in secondary school than in elementary school. Studies from Germany found in elementary schools stronger associations of SES with reading than with mathematics competencies. In a tracked school system like Germany, the relationship between grades and SES is weaker for secondary school than for elementary school students.

### 2.2. Intelligence and Scholastic Achievement

Intelligence is frequently considered the strongest predictor of academic performance (Kuncel et al. 2004). The effect of intelligence on test scores is stronger than its effect on grades (Lauermann et al. 2020). Correlations between general intelligence (*g*) and standardized test scores range from *r* = 0.61 to *r* = 0.77 (e.g., Koenig et al. 2008; Deary et al. 2007). Some studies find a slightly stronger association between standardized test scores and *g* for mathematics than for languages (e.g., Deary et al. 2007), while some find a weaker association for mathematics (O’Connell 2018) or similar correlations (e.g., Coyle 2015).

Intelligence and school grades correlated around *r* = 0.44 in a recent international meta-analysis (Roth et al. 2015), with no differences between mathematic/sciences grades and language grades. In high school and middle school, grades correlated significantly higher with *g* than grades in elementary school (Roth et al. 2015). However, in Germany with its tracked school system, a different pattern emerges. Here, intelligence was found to correlate more strongly with grades in mathematics than in native languages arts, both in elementary school (Schneider et al. 2018; Weber et al. 2013) and in secondary school (Steinmayr and Spinath 2008; Steinmayr et al. 2010).

### 2.3. Intelligence Level as a Moderator for the Link Between SES and Academic Achievement

While the overall impact of families’ SES on students’ achievement has been shown in many studies (e.g., OECD 2016; Reardon 2011; Sirin 2005), less is known about whether this relationship is the same across different levels of students’ ability. Previous research studying academic transitions assumed that socially advantaged families will actively support their children if they show bad or mediocre academic performances (Bernardi and Cebolla-Boado 2014; Bernardi and Triventi 2018; Gil-Hernández 2019), as they aim to place them in higher tracks notwithstanding their low achievement (Forster 2021), hence resulting in an interaction effect of family SES and achievement level on transitions. While this research mostly looked at students’ previous grades as an indicator of their ability (Bernardi and Cebolla-Boado 2014; Bernardi and Triventi 2018), a recent study using twin data from Germany analyzed whether the influence of families’ SES on transitions varied by their children’s intelligence (Gil-Hernández 2019). Contrary to expectations, high-SES families were not able to compensate for their child’s low performance at the bottom of the intelligence distribution. Overall, evidence on compensatory effects regarding transitions is mixed (Bukodi et al. 2014; Gil-Hernández 2019). While this strand of research gives important theoretical insights, the authors did not investigate whether intelligence might also moderate the link between SES and predictors of transition decisions, which is the focus of the present study. Transitions are highly related to grades and academic competency tests (Trautwein et al. 2006).

There is very little research on the influence of family SES, as differentiated by students’ ability level, on achieved competencies. The first empirical evidence from PISA 2015 (OECD 2016) suggests that the influence of a family’s SES is not the same at all levels of achievement. Looking at all OECD countries combined, the impact of families’ economic–social–cultural background on students’ science test scores was smaller for low-achieving students and high-achieving students, while that relationship was the most pronounced in students of average achievement. However, these analyses did not involve measures of students’ intelligence but used the PISA test score both for discerning the different achievement levels and for estimating the relationship between SES and achievement. Thus, the moderator and the dependent variable were the same, which does not meet the requirements of a moderation analysis (see Holland et al. 2017). We are not aware of a study that used a distinct performance indicator, such as intelligence, as a moderator of the link between SES and performance in academic competency tests.

However, there are rationales that the results found in the PISA 2015 sample (OECD 2016) might also be found in other samples. One might assume a lower association between SES and competencies in highly intelligent students, as they might be able to acquire knowledge and competencies at school with less parental support compared to averagely intelligent students since more intelligent people learn more easily and quickly than less intelligent people without support (see definition of intelligence by Neisser et al. 1996). Thus, the association between SES and academic achievement might be weaker in highly intelligent individuals. Also, in line with the above-cited PISA 2015 results, one might expect a diminished association in a below-average intelligence group. Given the strong linear relationship between intelligence and competency tests (Frey and Detterman 2004), it might be that a lower level of intelligence cannot be compensated by a more favorable learning environment when it comes to acquiring competencies as assessed in standardized scholastic competency tests. However, following the rationale of researchers investigating social mobility (e.g., Esping-Andersen and Cimentada 2018) with the claim that especially socially advantaged parents take great endeavors to compensate for their children’s lack of academic potential, it might also be that the link between SES and academic achievement is higher in low-intelligence groups.

Even less is known about a possible moderating effect of students’ intelligence on the impact of family SES on grades. However, comparable to competency tests, it might be that the families’ SES has a different impact on students’ grades depending on the child’s intelligence level. This effect might be even stronger than the effect for standardized competency tests, as grades encompass more than competency tests by being indicators of oral participation, effort, quality of homework, etc. (see Steinmayr et al. 2014). Average-intelligence students should be able to achieve above-average grades when putting forth effort and engagement. As school engagement and effective learning behaviors are associated with the students’ social background (Benner et al. 2016; Caro 2009; Roksa and Potter 2011), this is more likely for students of average intelligence from high-SES households than for those from low-SES households. Concerning students with below-average intelligence and socially advantaged parents, it might be that their lack of academic potential might be even more compensated by their parents, as they will be highly involved in order to prevent downward social mobility (e.g., Esping-Andersen and Cimentada 2018). This would result in an increased correlation between SES and grades in the low-intelligence group. However, at the same time, it is plausible that for students with below-average intelligence, grades might also vary less with their family’s SES, as good grades should require a minimum amount of academic potential. An apparent lack of subject-specific knowledge due to low intelligence might not be completely compensated by effective learning behaviors and support from home, which may result in less favorable grades partly irrespective of students’ SES-related support at home. This thought is supported by the aforementioned study by Gil-Hernández (2019), which found no advantage for children with low intelligence from higher-SES households.

Furthermore, it might also be that the relationship between SES and grades should also be smaller in the high-intelligence group. Highly intelligent students should be able to achieve good grades even without parental support in their schoolwork, as they are likely to understand most of the learning material at school without much difficulty or need for help. These thoughts are in line with a recent study that showed that more intelligent children are more likely to have better grades in school even if they are from low-SES families (Langensee et al. 2024). Additionally, high levels of parental support, which is more likely in high-SES families, will not improve highly intelligent children’s grades to the same extent as those of averagely intelligent children because there is less room to improve already fairly good grades given the linear relationship between intelligence and grades (see Roth et al. 2015).

### 2.4. Research Questions

The present study investigates whether the impact of SES on students’ achievement varies between different levels of student’s intelligence, using competency test scores and grades as achievement indicators in two school subjects, i.e., native language arts and mathematics, and in two samples, i.e., elementary school and secondary school students. As no other study has investigated this question before, and there are different streams of theoretical background leading to different conclusions, our analyses were rather exploratory. Based on the results of related studies and the theoretical thoughts outlined above, we propose the following research questions (RQ) for the comparison between the average and the above-average intelligence groups. Given the contradictory theoretical and empirical streams concerning the below-average intelligence group (increased (e.g., Esping-Andersen and Cimentada 2018) vs. diminished (e.g., OECD 2016) vs. unchanged (Gil-Hernández 2019) association between SES and academic achievement), we did not formulate any research questions concerning this group.

RQ 1: Is the relationship between elementary students’ school performance (grade and competency tests in both math and native language arts) and SES higher in the average intelligence (AI) group than in the above-average intelligence (AAI) group?
RQ2: Is the relationship between secondary school students’ school performance (grade and competency tests in both math and native language arts) and SES higher in the average intelligence (AI) group than in the above-average intelligence (AAI) group?

Since grades are based on both competencies and academic soft skills, it is plausible to assume that the proposed moderation might be stronger for grades than for competencies. While the relationships between SES and achievement seem to be similar in international studies in the verbal and the mathematical domains (e.g., OECD 2016), some German studies reported slightly stronger relationships between SES and grades in native language arts than grades in mathematics (e.g., Gröhlich and Guill 2009). This suggests the necessity of reporting and commenting on the moderation effects in these two different domains.

## 3. Method

### 3.1. Participants and Procedure

This study draws on the student data from a German research project on social inequality in school transitions (Fa(ir)bulous; Steinmayr et al. 2017) We re-analyzed both the elementary school and secondary school data from the Fa(ir)bulous study. As the secondary school sample did not cover all school tracks, it was extended (see also Bergold et al. 2020). Approval by an ethics committee was not required as per the institution’s guidelines and applicable regulations in the federal state in which the study was conducted. We received informed consent forms from the parents of all participating students, which were given by all parents since the present project was integrated into a larger school project for supporting students in making up their professional plans. Moreover, the responsive school administrations approved the study design and the data collection procedure beforehand. Participation was voluntary, and all present students agreed to participate.

Elementary school sample: A total of 837 fourth graders (424 boys and 411 girls; 2 students did not indicate their sex; mean age *M* = 9.14, *SD* = 0.54) from 56 classes from twenty elementary schools in different towns in North-Rhine Westphalia (Germany) participated in this project. A total of *n* = 483 of the children (58.0%) indicated that they mostly spoke German at home (four children did not indicate anything). Data were collected between September and December 2016 in the first term of fourth grade. We received parental informed consent from 1072 students (82% of all students located in the schools). However, fewer children were tested because many students were ill on the testing day, resulting in a final participation rate of 64%. Comparing the present data with those from the Ministry of Schools and Further Education NRW (2015) indicated that the participating children were representative of the population in the federal state where they came from in terms of gender ratio (χ^2^ = 0.002, *df* = 1, *p* = 0.960) and immigration background (χ^2^ = 0.063, *df* = 1, *p* = 0.802). Thus, we assume that the dropout due to illness did not influence the representativeness of the data.

Aside from variables that were assessed for the project Fa(ir)bulous, the variables of interest were assessed during regular class in students’ classrooms. All demographic variables were explained to the children and read aloud to make sure that all students worked at the same speed and that answers were not distorted by reading competencies. In total, the testing, including a break, took about two school lessons (95 min) and was conducted by trained research assistants.

Secondary school sample: The second sample consisted of the secondary school student sample of the project Fa(ir)bulous. As this sample did not include students from the highest academic track in the German school system (Gymnasium), additional data from a different project investigating Gymnasium students of the same age and using the same measures were included in our analyses. Thus, the secondary school sample comprised *N* = 2100 students (n = 1032 female, n = 1063 male, n = 5 students did not indicate their sex) in 9th and 10th grade from 22 schools (mean age: *M* = 15.31, *SD* = 0.74). Participation was voluntary and required informed consent by one parent. About 90% of all students attending the schools provided informed consent, and a total of 81% participated on the day of testing. The students attended one of the vocational track schools in Germany’s tracked school system (Hauptschule, the lowest vocational track: *n* = 229, 10.9%; Realschule, the higher vocational track: *n* = 669, 31.9%) or a Gymnasium, the academic track, (*n* = 722, 34.4%) or a comprehensive school (*n* = 480, 22.9%). Even though the comprehensive school is intended to cover all academic tracks, they should actually rather be considered as a vocational track school, as significant differences in student achievement and intelligence relative to the highest academic track have been documented in cross-sectional and longitudinal research (Guill et al. 2017; Lauermann et al. 2020). The distribution of students to school tracks was nearly identical to the distribution for the complete federal state for grades 9 and 10 (cf. Ministry of Schools and Further Education NRW 2016), with academic track schools slightly underrepresented and Realschule slightly overrepresented. About 37.4% of all students indicated that they themselves and/or at least one parent were either not born in Germany and/or that the student did not mostly speak German at home (ten students did not indicate anything on either of the variables).

### 3.2. Measures

Social background was assessed by means of a question on the number of books at home. This is a widely used indicator of parents’ cultural capital or home learning resources (e.g., Evans et al. 2010) that has been utilized in influential international large-scale assessments such as the Programme for International Student Assessment (PISA), the Trends in International Mathematics and Science Study (TIMSS), and the Progress in International Reading Literacy Study (PIRLS, e.g., Mullis et al. 2012). The number of books at home is typically used as an indicator of SES, with a higher number of books indicating a higher level of SES (e.g., Tan 2015). The variable primarily assesses a family’s objective cultural capital. Indirectly, it assesses the incorporated cultural capital, as the book variable correlates with a family’s educational level. It is an indicator that is frequently used in social research (cf. Heppt et al. 2022). Furthermore, it is frequently used in international competency studies and works equally well in samples of older and younger students (see Schwippert 2019). Schwippert (2019) demonstrated that the variable has excellent objective measurement properties independent from the focused sample (parent or student samples of different ages). This did not change even in this age of increasing digitalization, as analyses comparing the measurement properties of the book variable over time demonstrated (see ibid.). Furthermore, number of books at home also demonstrates strong correlations with other indicators of socioeconomic status (e.g., Eriksson et al. 2021), suggesting its overall suitability for measuring students’ socioeconomic background. Thus, the dichotomized book variable that we used has been demonstrated to be a very valid indicator of a family’s social background even though it is not perfect (for a criticism, see Engzell 2019). The item we used was identical to the one used in large-scale international scholastic achievement studies such as TIMSS or PIRLS and asked students “Approximately how many books are there in your home? (Do not count magazines, newspapers, or your schoolbooks.)”. Students indicated whether there were “None or very few (0–10 books)” (1), “Enough to fill one shelf (11–25 books)” (2), “Enough to fill one bookcase (26–100 books)” (3), “Enough to fill two bookcases (101–200 books)” (4), or “Enough to fill three or more bookcases (more than 200)” (5) (e.g., Wendt et al. 2016). For the elementary school sample, this was additionally illustrated by means of an image giving the children an idea about how many books were meant. Analogous to PISA (OECD 2005) and TIMSS (Stubbe et al. 2016), we created a dichotomous variable indicating whether a student lived in a household with more than a hundred books or less. This is a commonly applied cut-off value that separates homes with few or some resources vs. many learning resources (e.g., Mullis et al. 2012).

More than half (59.5%) of the students in the elementary school sample reported that they had 100 or fewer books at home. The characteristics of this sample approximate those of the nationally representative German elementary school sample from the PIRLS 2016 (Hußmann et al. 2017). In the secondary school sample, two-thirds of the sample (65.9%) indicated that they had 100 or fewer books at home, which also approximates the percentage indicating the same answer in a representative German sample of the same age (Sieben and Lechner 2019).

Grades of the elementary school sample: Schools provided copies of the report cards students received at the beginning of February 2017, i.e., the report card after the testing. These report cards indicated the students’ achievement in the first semester of the fourth grade and was the basis for the teachers’ recommendation for the secondary school track. By means of coding lists, report cards were matched to students’ assessment at the end of 2016. In Germany, grades range from 1 (“excellent”) to 6 (“insufficient/fail”). For a more intuitive understanding of the grades, all grades were recoded so that higher values denoted better grades.

Grades of the secondary school sample: All academic-track schools provided report cards of the last term that were matched with the students’ individual data. Data could be matched for about 99% of all academic-track students. The vocational-track school teachers provided information on students’ last report card grades in either native language arts (native language arts teacher) (response rate about 80%) or math (math teachers) (response rate about 85%). As in the elementary school sample, grades were recoded so that higher values denoted better grades.

Intelligence was assessed in both samples via the revised Culture Fair Test (CFT 20-R; Weiß 2006). This widely used intelligence test is based on the Culture Fair Test by Cattell and Cattell (1963) and assesses fluid intelligence, i.e., the ability to solve new problems independent of certain cultural skills, such as mathematics or reading competencies. We used the short form of the CFT-R, which consists of four subscales featuring a total of 56 items. Internal consistency was satisfactory (α = 0.76). The handbook provides standard norms that were used to determine students’ individual IQ value. The IQ score was recoded into below-average (IQ < 85), average (85 ≥ IQ < 115), and above-average (IQ ≥ 115) intelligence groups.

Mathematical competencies of the elementary school sample: One subtest of a German mathematical achievement test for third (second term) and fourth graders (first term) assessed students’ mathematical competencies (Deutscher Mathematiktest [DEMAT] 3+, Roick et al. 2004). The utilized subtest assessed arithmetic skills and was most strongly related to the total score (*r* = 0.89) among all subtests (see Roick et al. 2004). Internal consistency was good (α = 0.81).

Mathematical competencies of the secondary school sample: The test “Knowledge of Mathematical Conventions and Rules” (Konventions- und Regelwissen, KRW) consists of 50 items and is a subtest of the DEMAT 9 test (Schmidt et al. 2012), a widely used, validated, and normed test of mathematical competence in grade 9. Despite its short duration, it is a very good indicator of mathematical competence (Schmidt et al. 2012). Internal consistency was excellent (α = 0.94).

Reading competencies of the elementary school sample: One subtest of a native language arts reading achievement test for first through sixth grades was used to assess the students’ reading competencies (*ELFE 1–6. Ein Leseverständnistest für Erst- bis Sechstklässler*; Lenhard and Schneider 2006). We used the subtest “text comprehension”, which had a factor loading of γ = 0.91 on the more global construct of reading comprehension (see Lenhard and Schneider 2006). Internal consistency was good (α = 0.87).

Reading competencies of the secondary school sample: The LGVT 6–12 (*Lesegeschwindigkeits- und -verständnistest für die Klassen 6–12*; Schneider et al. 2007) is a valid, normed, and widely used test that measures reading comprehension in grades 6 to 12. Students have to read a continuous text with 1727 words and 23 blanks, for which they have to select the right word from three alternatives. Internal consistency was satisfactory (α = 0.78).

Covariates: As covariates, we assessed gender (dummy coded: female = 0; male = 1) and migration background (dummy coded: 0 = migration background, 1 = no migration background). Migration background was indicated by parents’ countries of birth and language primarily spoken at home. As data in the elementary school sample were mainly recruited in Rhine-Ruhr region, and as expected in this region, with its long history of labor immigration, students from immigrant backgrounds (as indicated by parents’ countries of birth and language primarily spoken at home) were overrepresented in relation to the elementary student population in all of NRW (49% vs. 38.3%; Ministry of Schools and Further Education NRW (2016)). The share of students from immigrant backgrounds in the secondary school sample was slightly higher than in the total student population in all of NRW (37.4% vs. 34.2%; Ministry of Schools and Further Education NRW (2016)).

### 3.3. Statistical Analysis

Moderator analyses were conducted with MPlus 8 (Muthén and Muthén 2017). We followed the syntax provided by Stride et al. (2015), which is based on a book by Hayes (2017). We used the syntax for model 1d (Stride et al. 2015, pp. 25–29) for a basic moderation with a categorial moderator. However, we made some changes, as the data were clustered. First, we used type = complex with school as a cluster variable. Second, we used maximum likelihood estimation with robust standard errors (MLR). Math and language arts grades as well as competency tests were separately regressed on the social background indicator (dummy variable “number of books”). The intelligence level (below average, average, and above average) served as the categorical moderator, which was represented by two dummy variables: Intelligence_dummy_1 = “Below-average intelligence” = 1, all others = 0; Intelligence_dummy_2 = “Above-average intelligence” = 1, all others = 0. The average intelligence group served as a reference group. The interaction terms were created by multiplying each intelligence dummy variable with the dummy variable “number of books”. In addition to the SES indicator, all achievement indicators were separately regressed on the two intelligence dummy variables as well as on the interaction terms. The model equation was as follows:

Y = b0 + b1X + b2WD1 + b3WD2 + b4×WD1 + b5×WD2

with Y = achievement indicator, X = dummy variable “number of books”, WD1 = Intelligence_dummy_1, WD2 = Intelligence_dummy_2, XWD1 = interaction term 1, and XWD2 = interaction term 2. This resulted in the following equations: 

The low-intelligence group: Y = (b0 + b2) + (b1 + b4) × X;

The high-intelligence group: Y = (b0 + b3) + (b1 + b5) × X;

The average-intelligence (reference) group: Y = b0 + b1× X.

Additionally, we ran moderator analysis with a continuous intelligence variable for the secondary school sample (cf. SOM, Table S2). Due to the fact that the elementary school sample performed better in the intelligence test better the norm calibration sample (cf. Section 4.1.2), we decided to run the analysis in the depicted way.

As school tracks, school performance, intelligence, and social background are strongly related in Germany (Lauermann et al. 2020), we controlled for school tracks to check whether intelligence and social background still have an impact on school performance when variance attributable to school tracks is accounted for. Thus, in the secondary school sample, we performed all analyses with and without controlling for the school track by means of a dummy variable contrasting Gymnasium, the academic track, with all other school non-academic school tracks (Hauptschule, Realschule, and Comprehensive School). This has been carried out in other studies as well (e.g., Becker et al. 2012) which has shown that only the school track Gymnasium had an impact when compared to other school tracks (see also Lauermann et al. 2020). Additionally, we checked whether further covariates or gender and immigration background would alter the results (cf. SOM, Tables S3 and S4).

## 4. Results

### 4.1. Results of the Elementary School Sample

#### 4.1.1. Descriptive Statistics of the Elementary School Sample

Means, standard deviations, and intercorrelations of all variables are displayed in Table 1. The mean score for the (categorized) number of books in the present sample was 3.29 (*SD* = 1.19), whereas in TIMSS/IGLU 2011, it was *M* = 3.17, *SD* = 1.16 (Wendt et al. 2016). Thus, the social background indicator was slightly higher than the one reported in TIMSS/PIRLS 2011, representing not even a small difference according to Cohen’s *d* (*d* = 0.102). The standard deviations were comparable. Furthermore, the average IQ of the present sample (*M* = 108.90; *SD* = 14.81) was above average, representing a medium effect (*d* = 0.592). However, the standard deviations of the IQ score were nearly identical in the norm and the present sample. The correlation between the dummy-coded variable “number of books” and reading and math competencies, respectively, were nearly identical to the ones found for Germany in PIRLS 2016 (*r* = 0.261; see Hußmann et al. 2017) and TIMSS 2015 (*r* = 0.195; Stubbe et al. 2016). The norm values for the competency tests based on the calibration sample were T = 48.82 (*SD* = 10.82) for reading competencies and T = 47.13 (*SD* = 11.00) for math competencies. Thus, reading and math competencies were a little bit beneath the calibration samples’ performance, whereas in both tests, the performance variance was a little higher.

As displayed in Table 1, the number of books positively correlated with all scholastic achievement measures and the intelligence score. The correlation between number of books and language arts grade was not higher than the one between number of books and math grade (*z* = 0.784, *p* = 0.432), but the correlations of number of books with reading and math competencies significantly differed (*z* = 2.204, *p* = 0.032). Reading competencies were more highly related to the number of books than were math competencies. Intelligence showed a stronger correlation with math grades than with language arts grades (*z* = 2.318, *p* = 0.020). The correlations between intelligence and each of the two competency tests did not differ (*z* = 0.733, *p* = 0.464).

#### 4.1.2. Moderator Analysis of the Elementary School Sample

Since the mean IQ of the sample was above average, fewer children were in the below-average IQ group (*n* = 51) than in the above-average group (*n* = 310). The largest group was the average intelligence group (*n* = 469). The norm sample was tested in 2002. Thus, the higher IQ of the present sample compared to the norm sample might be due to the Flynn effect (Flynn 1987). The Flynn effect covers an increase of about 3 IQ points every 10 years. As the present sample was tested about 15 years after the norm sample, 4.5 IQ-points of the difference between the norm and the present sample might be attributed to the Flynn effect. This might be especially true, as the results by Ang et al. (2010) indicated that the Flynn effect was highest in the 9-year-old age group, which is comparable to the present sample. We decided to retain the first recoding presented (below average: IQ < 85; average: 85 ≥ IQ < 115; above average: IQ ≥ 115) for the following reasons. First, we cannot rule out the option that the high intelligence of the investigated sample was completely attributable to sample specific characteristics (such as the slightly above-average socio-economic status) and not to the Flynn effect. Thus, categorizing them according to the intelligence distribution in the present sample, we would not be able to apply the results to the population, as we formulated our hypothesis with reference to a representative calibration sample; i.e., we formulated our hypotheses and research questions referring to below-average, average, and above-average intelligence groups in the respective population and not the given sample we investigated. Thus, we explicitly referred to population representative norms and categorized our samples accordingly. If we had categorized our sample based on the sample’s mean and standard deviation, students with above-average intelligence would have been categorized in the medium intelligence group, and students with average intelligence would have been categorized in the below-average intelligence group. Second, we wanted to apply the same coding for the elementary school sample and the secondary school sample so that in both samples, students were categorized based on their IQ scores and therefore with regard to the intelligence distributions of the respective populations (below average: IQ < 85; average: 85 ≥ IQ < 115; above average: IQ ≥ 115). The results of the moderator analysis are depicted in Table 2 and Table 3. Table 2 displays the interaction analyses, whereas Table 3 presents the conditional effects of social background on the achievement indicators.^1^

SES positively predicted academic achievement in all models. The variable “Intelligence 1”—coded as 1 = IQ < 85; 0 = average intelligence (85 ≥ IQ < 115) and above-average intelligence (IQ ≥ 115)—negatively predicted academic achievement in all models; i.e., belonging to the below-average intelligence group negatively predicted academic achievement. The variable Intelligence 2—coded as 1 = IQ ≥ 115 and 0 = 85 ≥ IQ < 115—positively predicted academic achievement; i.e., belonging to the above-average intelligence group predicted academic achievement positively. In RQ1, we postulated that the relationship between SES and students’ school performance would be higher in the average intelligence (AI) than in the above-average intelligence (AAI) group in the elementary school sample. Additionally, we tested whether the below-average intelligence (BAI) group differed from the other groups in their association between SES and academic achievement. The results only supported RQ1 when grades in native language arts (German) were used as a dependent variable. In line with the postulations in RQ1, the interaction between the AAI level and social background was significant when predicting language arts grades (cf. Table 2). Simple slope analyses (Table 3) demonstrated that, in the above-average intelligence group, the association between SES and language arts grades was lower than in the average intelligence group, even though it was still significant. The interaction term between BAI and SES when predicting language arts grades was not significant. Simple slope analyses demonstrated that SES and both grades were not significantly related in the BAI group, which is most likely due to the small sample size.

For all other dependent variables (math grades, math competencies, and reading competencies), we could not corroborate the assumptions made in RQ1 and RQ2, even though the interaction between the AAI level and SES slightly missed the significance level of *p* < 0.05 (*p* = 0.068) when predicting mathematical competencies (cf. Table 2). This was also reflected on a descriptive level in the simple slope analyses (Table 3), showing that SES had a stronger impact on mathematical competencies in the AI compared to the AAI group. The interactions for the below-average intelligence group were not significant for all dependent variables. Results did not change when additionally controlling for gender and migration background (see Table S5 Online Supplementary Material).

In summary, in the elementary school sample, we found some support for our research questions concerning the AAI group; i.e., language arts grades were more strongly associated with SES in the AI group than in the AAI group. No interaction terms concerning the BAI group were significant.

### 4.2. Results of the Secondary School Sample

#### 4.2.1. Descriptive Statistics of the Secondary School Sample

Means, standard deviations, and intercorrelations of all variables are displayed in Table 4. The mean score for the (categorized) number of books in the older sample was 2.96 (*SD* = 1.37). The average IQ of the older sample was *M* = 100.80 (*SD* = 15.35) and thus nearly identical to the norm sample. The average T-value for reading was *M* = 46.49 (*SD* = 10.80), and thus, the mean was slightly below the norm sample, while the variance was slightly increased. A slightly different picture was obtained for mathematical competencies, with an average T-value of *M* = 45.58 (*SD* = 9.01). Thus, the average mathematical performance was slightly below the mean of the norm sample, whereas the variance was slightly restricted.

As depicted in Table 4, number of books positively correlated with all scholastic achievement measures and the intelligence score. The correlation between the dummy-coded variable “number of books” and language arts grade and the one with math grades did not differ (*z* = 1.217, *p* = 0.224), which also applied to the correlations of the number of books with reading and math competencies (*z* = 0.213, *p* = 0.832). Intelligence showed a stronger correlation with math grades than with language arts grades (*z* = 4.222, *p* < 0.001). The correlations between intelligence and each of the two competency tests differed significantly (*z* = 5.261, *p* < 0.001). Mathematical competencies were more strongly related to intelligence than reading competencies.

#### 4.2.2. Moderator Analysis of the Secondary School Sample

The intelligence tests of *n* = 6 adolescents were missing for various reasons (urgent need to use the lavatory, getting sick during testing, and leaving testing early due to a different appointment). The rest were categorized as follows: below-average intelligence group (n = 293), average intelligence group (*n* = 1410), and above-average intelligence group (*n* = 391).^2^

Results of the moderator analysis are depicted in Table 5 and Table 6. Table 5 displays the interaction analyses with and without controlling for school track, whereas Table 6 presents the conditional effects of social background on the achievement indicators.

SES positively predicted academic achievement in all models. The variable “Intelligence 1”—coded as 1 = IQ < 85; 0 = average intelligence (85 ≥ IQ < 115) and above-average intelligence (IQ ≥ 115)—negatively predicted academic achievement in all models; i.e., belonging to the below-average intelligence group negatively predicted academic achievement. The variable Intelligence 2—coded as 1 = IQ ≥ 115 and 0 = 85 ≥ IQ < 115—positively predicted academic achievement; i.e., belonging to the above-average intelligence group predicted academic achievement positively. In RQ2, we postulated that the relationship between SES and students’ school performance would be higher in the average intelligence (AI) than in the above-average (AAI) group in the secondary school sample. The interaction between the AAI level and SES was significant when regressed on native language arts grades (cf. Table 5 and Table S2 in the Online Supplementary Material) when running the same analysis with a continuous intelligence variable. The relationship between secondary school students’ language arts grades and SES was stronger in the AI group than in the AAI group (cf. Table 6). This result did not change when controlling for school track. However, in the AAI group, SES was still significantly correlated with language arts grades, as displayed in Table 6. All other interaction terms were not significant (cf. Table 5). Thus, only one of the assumptions made for secondary school students was in line with the results. However, the different analyses with and without controlling for school track revealed that attending either an academic or a vocational school had quite a large impact on all school achievement indicators. The association with the dummy-coded school-track variable was more strongly associated with competency tests than with grades even after controlling for SES and intelligence. After controlling for SES, intelligence, and the interaction terms, the dummy-coded school-track variable incrementally explained 47.5% of the totally explained variance in reading and 57% in the math competency test, whereas it only incrementally explained 17% and 32%, respectively, of the total explained variance in language arts and math grades. The results did not change when additionally controlling for gender and migration background (see Table S6 Online Supplementary Material).

## 5. Discussion

While the relationship between families’ SES and students’ academic achievement is a well-established finding, the present study is the first to explore this relationship more deeply by taking the student’s level of intelligence into account. In the theoretical section, we introduced different views on how the intelligence level might moderate the association between SES and academic achievement. The overall assumption was that the association between SES and achievement would be strongest in students of average intelligence (AI), whereas the achievement of above-average intelligence (AAI) students should depend less on their families’ SES. Concerning the below-average intelligence group (BAI), no assumptions were made due to contradictory results and theories. The only consistent finding was that in both student groups, AAI students’ SES was less related to language arts grades than for their AI peers. In elementary school, the same interaction pattern slightly missed the significance level of *p* < 0.05 for math competencies. However, even though the associations were weaker in the AAI group, they were still significant in both samples, as the simple slope analyses revealed. These findings are remarkable in three ways.

First, SES was always an important predictor of both indicators of academic achievement (grades and standardized achievement tests) in both domains (language arts and math), which has already been demonstrated in various other studies (e.g., OECD 2019a; Steinmayr et al. 2012). The present study extends this finding by demonstrating that this is mostly irrespective of students’ intelligence level. To put it differently, students academically benefit from a home with advantageous SES at all levels of intelligence. This is a societal problem, as an adult’s social success depends on both their intelligence and educational success (see Ritchie and Bates 2013). Thus, even very intelligent children and adolescents from disadvantaged families are less likely to climb the social ladder, as they cannot unfold their academic potential as well as comparably intelligent students from more advantaged families (see also Deary et al. 2007; Forrest et al. 2011). Furthermore, less intelligent students from disadvantaged families run the risk of being doubly disadvantaged: They will probably struggle academically, as they lack the cognitive potential to easily master school’s requirements on their own. Additionally, they will probably not be supported by their parents as well as their socially advantaged peers of the same intelligence level, resulting in even more academic problems (see also Esping-Andersen and Cimentada 2018). Second, in both samples, the analyses yielded significant interactions when predicting language arts grades. These results suggest that the AI students benefit from high-SES background, especially for their grades in native language arts, more than the AAI students do, partly supporting the results by Langensee et al. (2024). Language arts skills like the quality of vocabulary and grammar strongly depend on the children’s background (e.g., Fernald et al. 2013; Perkins et al. 2013) and intelligence (Jensen 2002). Thus, it might be that highly intelligent children are more likely to develop language arts skills to an extent that is necessary for receiving good grades in language arts more independently from their family background compared to AI children. AI children may also reach the skill level necessary for good language arts grades, but they are more likely to reach this level if they possess a favorable socioeconomic background. However, even though the association was weaker in the highly intelligent student groups, language arts grades were still significantly related to SES in all groups in both samples, demonstrating that a high intelligence might buffer the association between SES and language arts grades but does not compensate it completely. This finding is alarming since teachers’ transition recommendations are based more strongly on language grades than on mathematics grades (Lehmann et al. 1997).

Third, we partly found slightly different results for elementary and secondary school. Whereas the interaction term with SES and the high-intelligence group in the prediction of math competencies slightly missed the significance level (*p* < 0.05) in the elementary school sample and might be significant in a larger sample, we found no effect in the secondary school sample. The math competencies of highly intelligent elementary children seem to develop more independently from their family background, but this effect was not present in the older sample. This might reflect that elementary school math is accessible for intelligent children without parental support, whereas less intelligent children depend more on their parents’ involvement with their schoolwork. At secondary level, as math becomes more complex and less self-evident, even highly intelligent children depend more strongly on resources at home.

Fourth, our results clearly demonstrate that cognitively challenged children having a below-average IQ did not benefit more from a socially advantageous background than their more intelligent peers with comparable social background. Thus, our results are in line with those found by Gil-Hernández (2019), who also found that a child’s background does not fully compensate for their lack of academic competencies in the low-intelligence group when transition decisions were considered. As the study by Gil-Hernández (2019) and the present study relied on their analyses of actual intelligence measures and not on proxies of intelligence, the combined results challenge the hypothesis that BA-intelligence children’s lack of academic potential might be even more compensated by their parents, as they will be highly involved in order to prevent downward social mobility (e.g., Esping-Andersen and Cimentada 2018). It might be that they are more involved than more intelligent children’s parents, but due to the strong association between intelligence and academic achievement (e.g., Lauermann et al. 2020), their enforced endeavors might be confined by their children’s lack of potential. For example, children’s crystallized intelligence (a construct highly related to scholastic competency tests) development was independent of students’ social background and cognitively activating environment at home (Schroeders et al. 2016). However, even though the association between SES and different academic achievement indicators is not stronger in the BA-intelligence group than in the other intelligence level groups, it was still significant. Thus, independent from their intelligence, children still academically benefit from homes with more favorable SES.

Other results are also worth discussing. In the present study, students’ reading competencies were more strongly related to SES than were math competencies (see also Hußmann et al. 2017 for PIRLs and Stubbe et al. 2016 for TIMSS). Thus, in elementary school, acquiring math competencies seems to be less dependent on a child’s social background than does the acquisition of reading competencies. This finding is in line with the view in educational research that, particularly in mathematics and science subjects, schools have “largely monopolized” (Baumert and Stanat 2006, p. 294) learning opportunities, while for the acquisition of reading skills, sufficient learning opportunities exist outside the classroom (Baumert and Stanat 2006), which depend on the family’s background. Given the overarching importance of reading competencies for acquiring further competencies in all subject areas as well as for successful participation in most areas of adult life (OECD 2019b), the stronger influence of SES on reading competencies in elementary school might further increase social inequalities. This was not the case in secondary school students (see also OECD 2019a), as also demonstrated by the secondary school sample in the present study. Here, SES was equally strongly related to academic achievement in both domains. Whether this is due to different reading competencies measures (the test in the secondary school had a stronger speed component) or really reflects different associations should be investigated in further studies. It is noteworthy that the overall associations between SES and competencies were stronger in secondary than in elementary school, which was also the case in other studies (see OECD 2019a in comparison to Hußmann et al. 2017; Wendt et al. 2016).

The differential effects found for elementary and secondary school students might be explained by the tracked secondary school system in Germany. Additionally, including the school track in the regression analyses increased the variance explanation in both grades and competency tests to a large extent even after controlling for SES and intelligence. It further decreased the association between SES and academic achievement. These findings mirror the finding that the placement into higher school tracks is related to students’ SES independently from achievement (Buchmann and Park 2009; Trautwein et al. 2006). In the federal state of Germany, where the study took place (as in most federal states), teachers give a transition recommendation based on a student’s former achievement, but in the end, parents decide which track their children will attend. When controlling for performance, high-SES parents send their children to the highest tracks more often than low-SES parents (Maaz et al. 2008). Thus, these low-performing (possibly also BAI) high-SES students then profit in their further competency development both from being exposed to the higher-quality, more challenging lessons at the Gymnasium and maybe additionally the support provided by their ambitious families. Earlier studies revealed that after controlling for intelligence, students in Germany showed differential achievement gains in mathematics as a function of the school track they attended, with larger gains in the academic tracks (Becker et al. 2006; Maaz et al. 2008). One might conclude that high-SES parents do their children a favor by placing their less intelligent children in higher tracks because even though they will not achieve good grades, they seem to develop relatively high competencies due to their more challenging (school and/or home) environment.

Another interesting finding is that both samples’ competency test performance was lower than what would have been expected based on their intelligence test. First, school performance (operationalized by both grades and test performance) does not only depend on intelligence but also on other student characteristics, such as motivation, personality traits, etc. (e.g., Steinmayr et al. 2019; Steinmayr and Spinath 2007). However, as both samples were randomly chosen, there is no reason to assume that theses students’ characteristics would be lower, which might explain the low test performance. Second, the state in which the sample was tested, North-Rhine Westphalia, consistently performs among the worst of all German federal states. The present study might be a hint that the educational system in this state hinders students from reaching the scholastic competencies that might be expected due to their cognitive potential.

However, the following limitations of the present study should be considered. First, the elementary school sample showed an above-average performance in the intelligence test. For this reason, it did not make sense to run interaction analyses with a continuous intelligence variable, as some students with above-average performances (according to the calibration norm sample) would have been considered as average-performing (in the present sample), and some average-performing students (according to the calibration norm sample) would have been considered as performing below the average (in the present sample). Categorizing them into three intelligence groups based on the calibration norm sample led to a reduction in variance in the multiple regression analysis testing for the interaction term. As most interaction terms were not significant, we additionally ran multiple regression analysis without an interaction term and intelligence as a continuous variable (cf. Tables S1 and S2 in the Online Supplementary Material), leading to more explained variance in the criteria. However, the simple slope analyses depicted in Table 3 and Table 6 would have been run in this way independent from the way we conducted the interaction analysis (based on continuous or non-continuous intelligence variables). Robinson et al. (2013) demonstrated that in comparison to testing the interaction term, testing the simple slopes for differences has increased power and less Type II error, even though the Type I error rates remain equivalent. Thus, the simple slope analysis depicted in Table 3 and Table 6 are merely a supplement to the conducted interaction analyses and clarify the direction of the interaction, i.e., whether the effect is stronger for the above-average, average, or below-average intelligence groups, a procedure that would have been identical if we had run all interaction analyses with intelligence as a continuous variable. Furthermore, as the intelligence distribution in the secondary school sample was nearly identical to the calibration norm sample, we could have also run interaction analysis with a continuous intelligence variable (cf. Table S4 in the Online Supplementary Material), leading to nearly identical results as in the analyses with the categorized intelligence variable.

Second, in line with the limitation that the elementary school sample’s intelligence distribution was above the average, the findings involving the below-average intelligence children in elementary school sample should be interpreted with caution, as this group was very small (n = 51). Thus, the lack of significant effects of social background on academic achievement revealed in this group by simple slope analyses should not be interpreted as evidence for lack of association. However, findings were largely unchanged when Flynn-effect-adjusted grouping was applied, yielding a somewhat larger group of BAI children (n = 81).

Third, in order to gain a better understanding of the relationship between family background and academic achievement, the present study focused exclusively on its possible variation between levels of intelligence. However, other personality traits are also important predictors of academic achievement (Steinmayr and Spinath 2008). Earlier research found that children’s temperament moderates the associations between SES-related risks and academic development (Wang et al. 2017), suggesting that family background does not have the same impact on all children. It might be fruitful to explore in future research whether students’ achievement is less dependent on their family background if they have very high levels of achievement motivation or conscientiousness compared to students scoring lower on these traits. Taking students’ motivation and personality traits, including intelligence, into account might also be a promising route for future research on the concept of (academic) resilience. Research on resilience has thus far given important insights into factors that enable children to succeed at school, even though they come from socially disadvantaged families. These factors include self-efficacy, planning, persistence (Martin and Marsh 2006), and supportive school communities (Borman and Overman 2004). However, the research on resilience has thus far predominantly focused on mediation and not moderation effects (e.g., Cappella and Weinstein 2001) and has paid very little attention to children’s intelligence.

A fourth limitation is the fact that indicators of SES and intelligence are confounded (cf. Strenze 2007). As we did not control for parental intelligence, we cannot separate the found effects from the possible effects of parents’ intelligence. We are not aware of any study investigating the impact of SES on children’s school performance while controlling for parental intelligence. However, parents’ specific competencies (biology and reading, respectively), the number of books, and the corresponding children’s competencies were investigated in two studies. In both studies, the number of books incrementally explained the variance in children’s competencies after controlling for parents’ respective competencies and further variables (Akukwe and Schroeders 2016; van Bergen et al. 2017). As reading competencies and science competencies, respectively, are highly correlated with intelligence (Rindermann 2006), one can cautiously interpret that the number of books explains the variance in children’s scholastic competencies that is not captured by parents’ intelligence.

Overall, our study has yielded mixed results. On the one hand, even when controlling for school track, students of all intelligence levels do profit from a higher-SES family background on all achievement indicators included in our analysis. Thus, students with a higher-SES family background will have better educational and job chances, as scholastic competencies predict future educational and vocational success independent from intelligence (Ritchie and Bates 2013). Apparently, students from low-SES families do not receive the necessary support from their schools to compensate for different scholastic familial support, as high-SES families are likely to have opportunities to compensate for the possible lack of school-relevant abilities in their children (Holzberger et al. 2023). This is a problem in general, as all students should be educated as well as possible irrespective of their social background (OECD 2019a). Additionally, highly intelligent low-SES students will possibly not aim at future achievements that they theoretically could achieve given their potential (Neubauer and Stern 2013). This highlights the societal problem of the inequality of opportunities for students from different social backgrounds. Schools should be ready to invest in programs to enrich the instruction of smart low-SES students, but so far, at least in Germany, this is not the case because at most schools, there are no enrichment programs for gifted children (Fischer and Müller 2014). On the other hand, we saw that these relationships were attenuated for the most intelligent children and adolescents on their grades in native language arts, as they were less dependent on their family background compared to students of average or low intelligence. No such differential effects were found for math grades. In summary, even high intelligence does not protect socially disadvantaged children from experiencing disadvantages in academic achievement. Given the importance of academic achievement for all indicators of occupational success (e.g., Ritchie and Bates 2013), this also constitutes a societal threat, as highly intelligent children from disadvantageous homes will be less likely to develop their full potential, even though our knowledge-oriented society needs their potential.

## Figures and Tables

**Table 1:** Means (M), Standard Deviations (SD), and Intercorrelations for All Investigated Variables; Elementary School Sample.

Variable	*M*	*SD*	2	3	4	5	6
1 Number of books ^a^	3.29	1.19	0.305 **	0.284 **	0.243 **	0.193 **	0.274 **
2 Native language arts grade ^b^	4.35	0.88		0.667 **	0.426 **	0.452 **	0.625 **
3 Math grade ^b^	4.28	0.99			0.483 **	0.592 **	0.443 **
4 Intelligence IQ	108.90	14.81				0.461 **	0.437 **
5 Mathematical competencies ^c^	8.36	3.49					0.392 **
6 Reading competencies ^d^	12.15	4.57					

*Note*. *N* = 800–838. ^a^ Range: 1 to 5. ^b^ Range: 1 to 6. ^c^ Range: 0 to 15. ^d^ Range: 0 to 20. ** *p* < 0.01.

**Table 2:** Standardized Results of Moderator Analyses Regressing Academic Achievement Indicators in Mathematics and Native Language Arts on Dummy Variables of Number of Books, Intelligence Groups, and their Interaction Terms in the Elementary School Sample.

	Grades				Standardized Achievement Tests			
Variable	B		*SE*		B		*SE*	
Domain	NLA	Math	NLA	Math	NLA	Math	NLA	Math
Number of books ^a^	0.267 ***	0.217 ***	0.044	0.045	0.180 ***	0.141 ***	0.047	0.046
Intelligence 1 ^a^	−0.152 ***	−0.125 ***	0.026	0.029	−0.157 ***	−0.241 ***	0.026	0.037
Intelligence 2 ^a^	0.369 ***	0.395 ***	0.033	0.041	0.321 ***	0.335 ***	0.037	0.038
Number of books × Intelligence I	−0.022	−0.046	0.043	0.038	−0.016	0.014	0.036	0.037
Number of books × Intelligence II	−0.128 **	−0.053	0.047	0.053	−0.020	−0.095 #	0.053	0.053
R^2^	0.198	0.232	0.022	0.025	0.190	0.186	0.028	0.028

# *p* ≤ 0.10, ** *p* ≤ 0.01, and *** *p* ≤ 0.001. ^a^ Dummy coded. NLA = native language arts; Intelligence 1: 1 = below-average intelligence (IQ < 85), 0 = average intelligence (85 ≥ IQ < 115), and above-average intelligence (IQ ≥ 115); Intelligence 2: 1 = above-average intelligence (IQ ≥ 115), 0 = average intelligence (85 ≥ IQ < 115), and below-average intelligence (IQ < 85).

**Table 3:** Conditional Unstandardized Effect of Social Background on Academic Achievement Indicators in Mathematics and Native Language Arts in Groups Defined by Intelligence Group; Elementary School Sample.

	Grades				Standardized Test Performance			
	B		*SE*		B		*SE*	
Intelligence Group	NLA	Math	NLA	Math	NLA	Math	NLA	Math
Below Average	0.321	0.064	0.304	0.311	1.346	1.386	1.436	0.908
Average	0.479 ***	0.437 ***	0.081	0.092	1.869 ***	1.004 **	0.486	0.329
Above Average	0.190 *	0.304 ***	0.080	0.080	1.018 **	0.143	0.375	0.289

*Note*. NLA = native language arts; Below Average: IQ < 85; Average: 85 ≥ IQ < 115; Above Average: IQ ≥ 115. * *p* ≤ 0.05, ** *p* ≤ 0.01, and *** *p* ≤ 0.001.

**Table 4:** Means (*M*), Standard Deviations (*SD*), and Intercorrelations for All Investigated Variables; Secondary School Sample.

Variable	*M*	*SD*	2	3	4	5	6
1. Number of books ^a^	2.96	1.37	0.239	0.208	0.284	0.363	0.358
2. Native language arts grade ^b^	3.95	0.92		0.438	0.210	0.267	0.276
3. Math grade ^b^	3.89	1.07			0.316	0.433	0.204
4. Intelligence IQ	100.80	15.35				0.465	0.345
5. Mathematical competencies ^c^	19.85	9.90					0.465
6. Reading competencies ^d^	10.51	5.89					

*Note*. *N* = 1624–2083. ^a^ Range: 1 to 5. ^b^ Range: 1 to 6. ^c^ Range: 0 to 50. ^d^ Range: 0 to 23. All correlations significant at *p* < 0.001.

**Table 5:** Standardized Results of Moderator Analyses Regressing Academic Achievement Indicators in Mathematics and Native Language Arts on Dummy Variables of Number of Books, Intelligence Groups, and their Interaction Terms with and Without Controlling for School Types; Secondary School Sample.^3^.

	Grades				Standardized Achievement Tests			
Variable	B		*SE*		B		*SE*	
Domain	NLA	Math	NLA	Math	NLA	Math	NLA	Math
Number of books ^a^	0.221 ***	0.161 ***	0.032	0.032	0.234	0.233	0.028	0.034
Intelligence 1 ^a^	−0.121 ***	−0.108 ***	0.025	0.025	−0.168	−0.236	0.030	0.031
Intelligence 2 ^a^	0.121 ***	0.234 ***	0.026	0.029	0.178	0.269	0.036	0.038
Number of books × Intelligence I	−0.007	−0.004	0.029	0.030	0.029	0.032	0.024	0.019
Number of books × Intelligence II	−0.083 ***	−0.057	0.030	0.051	−0.007	−0.056	0.035	0.042
R^2^	0.075	0.094	0.012	0.017	0.148	0.211	0.029	0.031
Controlling for School Type								
	Grades				Standardized Achievement Tests			
Variable	B		*SE*		B		*SE*	
Domain	NLA	Math	NLA	Math	NLA	Math	NLA	Math
Number of books ^a^	0.181 ***	0.095 **	0.026	0.028	0.122 ***	0.071 **	0.026	0.022
Intelligence 1 ^a^	−0.109 ***	−0.090 ***	0.026	0.023	−0.135 ***	−0.188 ***	0.026	0.029
Intelligence 2 ^a^	0.102 ***	0.199 ***	0.026	0.031	0.120 ***	0.184 ***	0.025	0.031
Number of books × Intelligence I	−0.009	−0.009	0.029	0.027	0.022	0.022	0.025	0.014
Number of books × Intelligence II	−0.082 **	−0.047	0.029	0.052	0.011	−0.032	0.032	0.035
School Type ^a^	0.133 ***	0.224 ***	0.040	0.048	0.389 ***	0.564 ***	0.037	0.046
R^2^	0.090	0.138	0.016	0.030	0.282	0.490	0.041	0.055

*Notes.* ** *p* ≤ 0.01, *** *p* ≤ 0.001. ^a^ Dummy coded. NLA = native language arts; Intelligence 1: 1 = below-average intelligence (IQ < 85), 0 = average intelligence (85 ≥ IQ < 115), and above-average intelligence (IQ ≥ 115); Intelligence 2: 1 = above-average intelligence (IQ ≥ 115), 0 = average intelligence (85 ≥ IQ < 115), and below-average intelligence (IQ < 85).

**Table 6:** Conditional Unstandardized Effect of Social Background on Academic Achievement indicators in Mathematic and NLA in Groups Defined by Intelligence Group; Secondary School Sample.

	Grades				Standardized Test Performance			
	B		*SE*		B		*SE*	
Intelligence Group	NLA	Math	NLA	Math	NLA	Math	NLA	Math
Below Average	0.380 *	0.333	0.191	0.224	4.137 ***	7.113 ***	1.114	1.726
Average	0.426 ***	0.367 ***	0.062	0.075	2.914 ***	4.868 ***	0.390	0.855
Above Average	0.159 *	0.156	0.067	0.180	2.778 ***	2.947 **	0.569	1.074

*Note*. NLA = native language arts; Below Average: IQ < 85; Average: 85 ≥ IQ < 115; Above Average: IQ ≥ 115. * *p* ≤ 0.05, ** *p* ≤ 0.01, and *** *p* ≤ 0.001.

## Data Availability

The raw data supporting the conclusions of this article will be made available by the authors on request.

## Notes

1 We performed an alternative recoding of the IQ scores controlling for the possible Flynn effect: below average (IQ < 89.5), average (89.5 ≥ IQ > 119.5), and above average (IQ ≥ 119.5). The result pattern for the moderator analysis when the Flynn-effect-adjusted coding was used did not differ;Categorizing the intelligence variable led to some restriction in variance in this variable, which resulted in less explained variance in the achievement criteria in the elementary school sample. As most interaction terms were not significant, we additionally present multiple regression analysis with SES and intelligence as a continous variable regressed on the different achievement criteria in the Online supplement material (Tables S1 and S2).

2 Moderator analyses were also run with intelligence as a continuous variable (see Online Supplementary Material Table S4). The results did not change.

3 Table S3 in the Online Supplementary Material shows the same analysis with a further indicator of socioeconomic status: parents’ highest school leaving certificate. The results were replicated, which demonstrates their robustness.

## Supplementary Materials

The following supporting information can be downloaded at https://www.mdpi.com/article/10.3390/jintelligence12120123/s1: Table S1: Standardized Results of Analyses Regressing academic achievement indicators in Mathematics and German on Dummy Variables of number of books and Intelligence as a continuous variable with Interaction Terms (Elementary School Sample) (p. 2); Table S2: Standardized Results of Analyses Regressing academic achievement indicators in Mathematics and German on Dummy Variables of number of books and Intelligence as a continuous variable with Interaction Terms (Secondary School Sample) (p. 3); Table S3: Additional analyses conducted with the Secondary School Sample (p. 4); Table S4: Results of an alternative moderation analysis with a continuous intelligence variable for the secondary school sample (p. 5); Tables S5 and S6: Moderation analysis controlling for further variables (pp. 6–7).
