# Supplementary material for: Does Students’ Level of Intelligence Moderate the Relationship Between Socio-Economic Status and Academic Achievement?
Source: J Intell. 2024 Dec 1;12(12):123. doi: 10.3390/jintelligence12120123 (PMC11727768; doi:10.3390/jintelligence12120123)
Supplement: Supplementary file 1 [file jintelligence-12-00123-s001.zip › jintelligence-3129737-supplementary.pdf]

Supplemental Material

Does Students' Level of Intelligence Moderate the Relationship Between Socio-Economic Status and Academic Achievement?

Journal of Intelligence

This supplementary file provides an overview of the

(1) Table S1: Standardized Results of Analyses Regressing academic achievement indicators in Mathematics and German on Dummy Variables of number of books and Intelligence as a continuous variable with Interaction Terms (Elementary School Sample) (p. 2);

(2) Table S2: Standardized Results of Analyses Regressing academic achievement indicators in Mathematics and German on Dummy Variables of number of books and Intelligence as a continuous variable with Interaction Terms (Secondary School Sample) (p. 3);

(3) Table S3: Additional analyses conducted with the Secondary School Sample (p. 4);

(4) Table S4: Results of an alternative moderation analysis with a continuous intelligence variable for the secondary school sample (p. 5);

(5) Table S5-S6: Moderation analysis controlling for further variables (pp. 6-7).

Table S1

Standardized Results of Analyses Regressing academic achievement indicators in Mathematics and German on Dummy Variables of number of books and Intelligence as a continuous variable with Interaction Terms (Elementary School Sample)

	Grades				Standardized Achievement Tests			
Variable	*B*		*SE*		*B*		*SE*	
Domain	NLA	Math	NLA	Math	NLA(R)	Math	NLA(R)	Math
Number of books	.183***	.166***	.034	.031	.146***	.077**	.032	
Intelligence	.399***	.458***	.025	.027	.412***	.448***	.033	
R^2	.214	.260			.212	.218		

Notes. # p ≤ .10, * p ≤ .05, ** p ≤ .01, *** p ≤ .001. NLA(R) = native language arts (Reading competencies); [a]Dummy coded.

Table S2

Standardized Results of Analyses Regressing academic achievement indicators in Mathematics and German on Dummy Variables of number of books and Intelligence as a continuous variable with Interaction Terms (Secondary School Sample)

	Grades				*Standardized Achievement Tests*			
Variable	*B*		*SE*		*B*		*SE*	
Domain	NLA	Math	NLA	Math	NLA(R)	Math	NLA(R)	Math
Number of books	.182***	.125***	.026	.030	.226***	.202***	.022	.025
Intelligence	.172***	.288***	.023	.024	.295***	.412***	.036	.031
R^2	.077	.115			.169	.250		

Notes. # p ≤ .10, * p ≤ .05, ** p ≤ .01, *** p ≤ .001. NLA(R) = native language arts (Reading competencies); [a]Dummy coded.

Table S3

Standardized Results of Moderator Analyses Regressing academic achievement indicators in Mathematics and German on Dummy Variables of student reported parents' highest school leaving certificate (academic vs. vocational track), Intelligence groups and their Interaction Terms Secondary School Sample.

	Grades				*Standardized Achievement Tests*			
Variable	*B*		*SE*		*B*		*SE*	
Domain	NLA	Math	NLA	Math	NLA	Math	NLA	Math
HSLC	.159***	.160***	0.046	0.034	.171***	.229***	0.044	0.047
Intelligence 1[a]	-.129***	-.081**	0.029	0.028	- .164***	-.217***	0.034	0.038
Intelligence 2[a]	.134***	.257***	0.039	0.038	.230***	.283***	0.038	0.039
HSLC x Intelligence I	-0.024	-.052*	0.031	0.025	- .024	-.048	0.033	0.033
HSLC x Intelligence II	-0.087**	.064#	0.033	0.036	- .054	-.023	0.048	0.043
R^2	.056	.093			.117	.224		

Notes. # p ≤ .10, * p ≤ .05, ** p ≤ .01, *** p ≤ .001. HSLC = Parents' Highest School Leaving Certificate; NLA = native language arts; [a]Dummy coded. Intelligence 1: 1 = below average intelligence (IQ < 85) , 0 = average intelligence (85 ≥ IQ > 115) and above average intelligence (IQ ≥ 115); Intelligence 2: 1 = above average intelligence, 0 = average intelligence and below average intelligence.

Table S4

Standardized Results of Moderator Analyses Regressing academic achievement indicators in Mathematics and German on Dummy Variables of number of books, Intelligence as a continuous variable and their Interaction Terms for above average students in the Secondary School Sample.

	Grades				*Standardized Achievement Tests*			
Variable	*B*		*SE*		*B*		*SE*	
Domain	NLA	Math	NLA	Math	NLA(R)	Math	NLA(R)	Math
Number of books	.209***	.128***	.030	.030	.227***	0.217***	.024	.028
Intelligence	.201***	.292***	.021	.024	.296***	.429***	.036	.032
Interaction term	-.075***	-.010	.029	.047	-.004	-.042	.028	.031
R^2	.080	.115			.169	.251		

Notes. # p ≤ .10, * p ≤ .05, ** p ≤ .01, *** p ≤ .001. NLA(R) = native language arts (Reading competencies); [a]Dummy coded. Interaction term: Number of books * above average intelligence (IQ > 15).

Table S5

Standardized Results of Moderator Analyses Regressing academic achievement indicators in Mathematics and Native Language Art on Dummy Variables of Number of Books, Intelligence groups, and their Interaction Terms Elementary School Sample with covariates.

Analyses with covariates								
	Grades				*Standardized Achievement Tests*			
Variable	*B*		*SE*		*B*		*SE*	
Domain	German	Math	Germ.	Math	German	Math	Germ.	Math
Number of books[a]	0.253***	0.205***	0.045	0.047	0.184***	0.139**	0.051	0.046
Intelligence 1[a]	-0.138***	-0.135***	0.026	0.030	-0.177***	-0.238***	0.035	0.039
Intelligence 2[a]	0.345***	0.383***	0.034	0.042	0.314***	0.331***	0.038	0.039
Number of books x Intelligence I	-0.037	-0.040	0.042	0.038	-0.029	0.015	0.042	0.038
Number of books x Intelligence II	-0.128**	-0.038	0.046	0.052	-0.069	-0.089#	0.050	0.054
Migration background[a]	0.146***	0.006	0.040	0.034	0.102**	-0.027	0.033	0.037
Gender[b]	-0.187***	0.096***	0.026	0.025	-0.129***	0.028	0.029	0.031
R^2	.250	.407			.215	.180		

$p \leq .10$, * $p \leq .05$, ** $p \leq .01$, *** $p \leq .001$. [a]Dummy coded: 0 = migration background, 1 = no migration background. [b] female = 0; male = 1. Intelligence 1: 1 = below average intelligence, 0 = average intelligence and above average intelligence; Intelligence 2: 1 = above average intelligence, 0 = average intelligence and below average intelligence.

Table S6

Standardized Results of Moderator Analyses Regressing academic achievement indicators in Mathematics and Native Language Art on Dummy Variables of Number of Books, Intelligence groups, and their Interaction Terms Secondary School Sample with covariates.

	Grades				*Standardized Achievement Tests*			
Variable	*B*		*SE*		*B*		*SE*	
Domain	German	Math	Germ.	Math	German	Math	Germ.	Math
Number of books[a]	0.175***	0.094**	0.023	0.028	0.097***	0.070**	0.024	0.021
Intelligence 1[a]	-0.100***	-0.088***	0.026	0.023	-0.122***	-0.192***	0.024	0.029
Intelligence 2[a]	0.112***	0.201***	0.022	0.030	0.113***	0.177***	0.023	0.030
Number of books x Intelligence I	-0.019	-0.010	0.027	0.027	0.016	0.025	0.025	0.034
Number of books x Intelligence II	-0.087**	-0.046	0.034	0.055	0.010	-0.025	0.031	0.034
School Track[a]	0.081*	0.215***	0.033	0.049	0.326***	0.576***	0.036	0.044
Migration background[b]	0.083**	0.016	0.028	0.025	0.180***	-0.017	0.024	0.025
Gender[c]	-0.243***	-0.023	0.020	0.031	-0.007	0.103***	0.024	0.025
R^2	.154	.140			.308	.501		

Notes. # $p \leq .10$, * $p \leq .05$, ** $p \leq .01$, *** $p \leq .001$. [a]Dummy coded: 0 = vocational track, 1 = academic track. [b]Dummy coded: 0 = migration background, 1 = no migration background. [c] female = 0; male = 1. Intelligence 1: 1 = below average intelligence, 0 = average intelligence and above average intelligence; Intelligence 2: 1 = above average intelligence, 0 = average intelligence and below average intelligence.
